# Radon emission fluctuation as a result of biochar application into the soil

**DOI:** 10.1038/s41598-021-93277-7

**Published:** 2021-07-05

**Authors:** Kamil Szewczak, Sławomir Jednoróg, Katarzyna Wołoszczuk, Łukasz Gluba, Anna Rafalska-Przysucha, Mateusz Łukowski

**Affiliations:** 1 Institute of Agrophysics, Polish Academy of Sciences, Doświadczalna 4, 20-290 Lublin, Poland; 2 Central Laboratory for Radiological Protection, Konwaliowa 7, 01-194 Warsaw, Poland

**Keywords:** Natural hazards, Agroecology

## Abstract

The presented research was focused on the analysis of the impact of biochar application into the soil on the radon exhalation process as a new issue of radiation protection in agriculture. Field measurements of the radon exhalation rate utilizing two methods—active and passive as well as laboratory measurements of the radon emanation coefficient were performed. In laboratory a soil samples with sunflower husk biochar were analysed using the accumulation chamber technique. At the final step the assessment of the effective dose for humans coming from radon exhalation from soil depending on biochar dose applied were evaluated. The doses of biochar applied in the analysed experimental fields were 0, 20, 40, 60, 80, and 100 Mg ha^−1^. The results show that biochar application into the soil contribute to a decrease in the emanation coefficient from a value around 7% to less than 2% with a simultaneous decrease in the radon exhalation rate from 4.4 to 14.8 mBq m^−2^ s^−1^ when the biochar dose increase from 0 to 100 Mg ha^−1^.

## Introduction

Radon is a radioactive gas occurs in the environment as a result of the decay of radium (Ra-226) present in the geological base of the soil. The radon emission from the soil can be parameterized by two processes: radon emanation and radon exhalation. Radon emanation, commonly expressed as an emanation coefficient ε (in %), describes the amount of radon particles that are able to escape from soil grains into the free air-filled space in the soil structure. The radon exhalation rate ERn (expressed in mBq m^−2^ s^−1^) describes the amount of radon gas present on the ground surface. The amount of radon present in the soil air depends on the activity concentration of Ra-226 in the examined region, characteristic geological soil properties of the region^1^, and the mode of soil use^2^. Currently in compliance with the EU Directive, the specific radon hazardous regions in Europe are specified and the problem of radon exposure for general population was highlighted. The main reason for these activities is the fact that radon is the main factor of human exposure to ionizing radiation from natural sources^3–6^. The total annual effective dose for humans from radon inhalation in buildings may reach values above 8 mSv per year^7^. As proved before, the radon emission from the ground surface is governed by complex processes and is dependent on many factors^8–10^. In regions that are not influenced by human activity, such as forests or agricultural wasteland, radon emissions from the soil surface depend mostly on geological characteristics^11^ and soil moisture^12^. However, in agricultural regions, additional factors have to be taken into account when the process of radon emission from soil is considered. The impact of tillage practices and soil fertilization should at least be considered^12^. In addition the amount of the radon released into the environment is a one of the factor combined into the mathematical model to assess humans exposure for indoor radon^13^.

Modern, sustainable agriculture is constantly looking for materials to improve cultivation conditions while having a neutral impact on the environment. One of such materials is biochar^14^. Biochar is a modern materials examined continuously in terms of its use as a fertilizer in sustainable agriculture^15,16^. It is produced in the process of pyrolysis of organic input material, e.g. wood chips^17^, coconut husk^18^, or sunflower husk^19^. The main research trends focused on the application of biochar in agriculture include improvement of soil water retention^20^, reduction of heavy metal pollution in soil^21^, and improvement of the fertilization process^22^. Biochar is a porous material characterized by extremely high specific surface area^23^ and is a completely natural supplementation material. Recently, the ability of biochar to remove radioactive material from soil has been investigated^24^. The impact of biochar on environmental radioactivity has been reported recently^25^ showing that biochar application into the soil even if not reducing a natural and anthropogenic radioisotopes activity concentration it could influence for radon emission process from soil. Even if the critical review for summarize the impact of biochar application into the soil was performed the assessment for radon emission was not analysed in details yet^14^. The biochar is currently commercially used in agriculture in such a countries as Australia^26^, United State of America^27^ and China^28^. In Poland the research on the biochar application for purpose of improve the soil water retention properties are in progress^29^. For purpose of agriculture the typically biochar dose applied into the soil ranged from 10 to 100 Mg ha^−1^^22,30,31^.

As the preliminary research proved the impact of biochar for radon emission from soil^25^ at the presented work we go into the details by showing the direct influence of the biochar for two parameters describing the radon emission from soil—the exhalation rate and the emanation coefficient and assess how the biochar application could influence on radon presence in air at bigger scale.

In the present study, we analyse the impact of biochar on radon emanation and radon exhalation rates based on in-field measurements and examination samples in laboratory. The aim of the research was to elucidate the effect of biochar produced from sunflower husk applied into the soil at five specific doses on the radon emanation and radon exhalation processes. Basing on the performed research we present the impact of the biochar application into the soil for state of the environment in the context of the radon emission. Taking into account the presented results and the literature data we investigate the perspectives for changes of outdoor radon emission for specific agro area where the biochar was applied around the world.

## Methods

In order to study the impact of biochar application into the soil for the emanation coefficient and radon exhalation rate, biochar was applied to the soil in various doses. Then, after the soil stabilization period, samples were taken for laboratory tests and field measurements of exhalation arte were carried out. Based on the obtained results, a discussion was conducted focused on the perspective of environmental changes in the context of radon emission from soil depending on the dose of biochar. A detailed description of the materials and measurement methods used to conduct the research is presented in the following subsections.

### Biochar

The biochar incorporated in the field experiment was produced from sunflower husk in the pyrolysis process in the temperature range of 450–550 °C and consist on grains with diameters from 50 μm up to 10 mm. The biochar was characterized by specific surface area of 2.0 m^2^ g^−1^ in occupancy of Cu ions and 5.1 m^2^ g^−1^ for Ag ions^23^.

### Field experiment

The field experiment was conducted in ten plots, each with a dimension of 1.1 m × 1.1 m, located in Lublin/Felin at the Institute of Agrophysics of Polish Academy of Science. In addition one plot was left without biochar as a reference. The biochar was applied into the soil in April 2018. The soil presented on the fields were classified as Haplic Luvisol with 66% of sand, 23% of silt 11% of clay and 0.91% of organic matter (data for 0–15 cm layer)^32^. The following doses of biochar were applied for the following fields: 1, 5, 10, 20, 30, 40, 50, 60, 80, and 100 Mg ha^−1^ (which corresponded to the percentage of biochar per unit mass of soil: 0.05, 0.24, 0.48, 0.95, 1.43, 1.90, 2.28, 2.86, 3.81, and 4.76%, respectively). The fields were kept without vegetation by application of herbicide (Roundup 360 PLUS at 2.5 L ha^−1^). For the purpose of presented work six fields were investigated: with 0 (control), 20, 40, 60, 80 and 100 Mg ha^−1^ biochar doses.

### Soil sample collection and preparation

**Figure 1:**
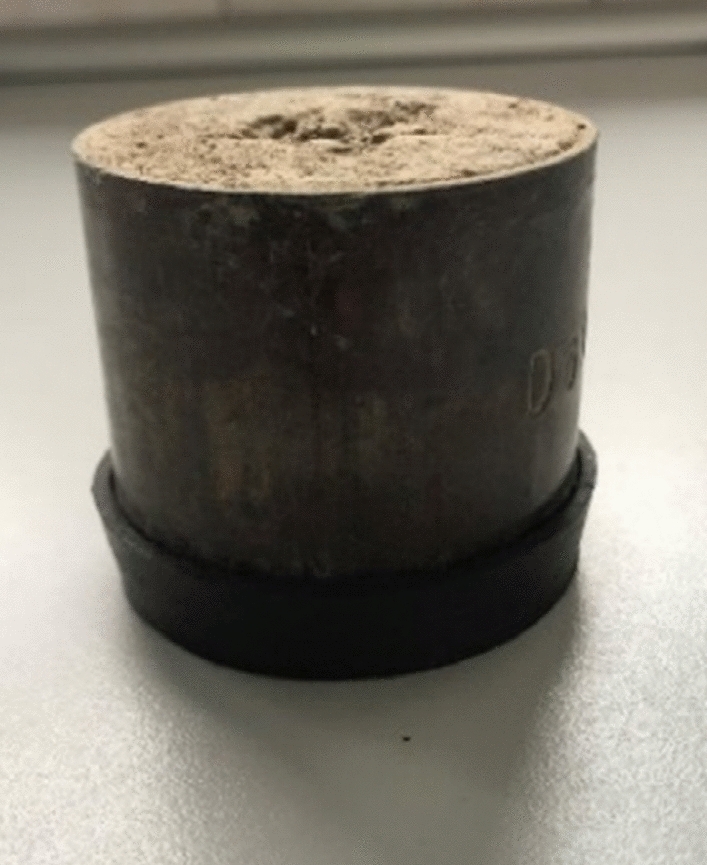
The example of sample collected from the experimental field and prepared for measurements within the small accumulation chamber in the process of radon emanation assessment.

The soil samples were collected from five experimental plots, where the biochar was applied into the soil at the doses of 20, 40, 60, 80, and 100 Mg ha^−1^, and from a control field, where no biochar was applied (denoted as 0 Mg ha^−1^). Soil samples were collected from each field at five statistically chosen points to get about 2 kg of soil. After collection, the soil was mixed and dried at room temperature for two weeks. One sample for laboratory examinations was prepared from each part of the soil. The sample was a 4.7 cm high and 5.2 cm diameter steel cylinder, as presented in Fig. 1. The volume of each sample was 100 cm^3^. The bulk density of each sample was evaluated. The soil net weight of each sample was measured by measurements of each sample and subtraction of the weight of the steel cylinder. Next, the cylinders were closed on the bottom with a rubber cap to reduce the radon emanation surface to the size of 19.62 cm^2^ at the top of the sample.

### Total porosity measurements

The total porosity was measured with the weight method after water saturation. First, the samples were dried at 105 °C for 1 h and weighed to measure the total mass of the soil-biochar samples. To assess the total pore volume, the samples were placed in a tray filled with water for 24 h to reach saturation and the weight measurements were repeated. The total porosity η was calculated according to the equation:

$$\eta =\frac{{V}_{p}}{{V}_{s}}=\frac{{m}_{w}}{{\rho }_{w}\cdot {V}_{s}}=\frac{\left({m}_{s}-{m}_{d}\right)}{{\rho }_{w}\cdot {V}_{s}}$$

where m_s_ is the mass of a saturated sample, m_d_ is the mass of a dried sample, V_s_ and V_p_ represent the sample volume (100 cm^3^) and pore volume, respectively, and ρ_w_ is water density. The weight measurements were realized by electronic laboratory balance with the accuracy of 0.1 mg. The total uncertainty of the method was assessed for 5%.

The uncertainty for radon in air concentration measurements were assessed for 18% basing on the data provided by the instrument.

### Emanation coefficient assessment

**Figure 2:**
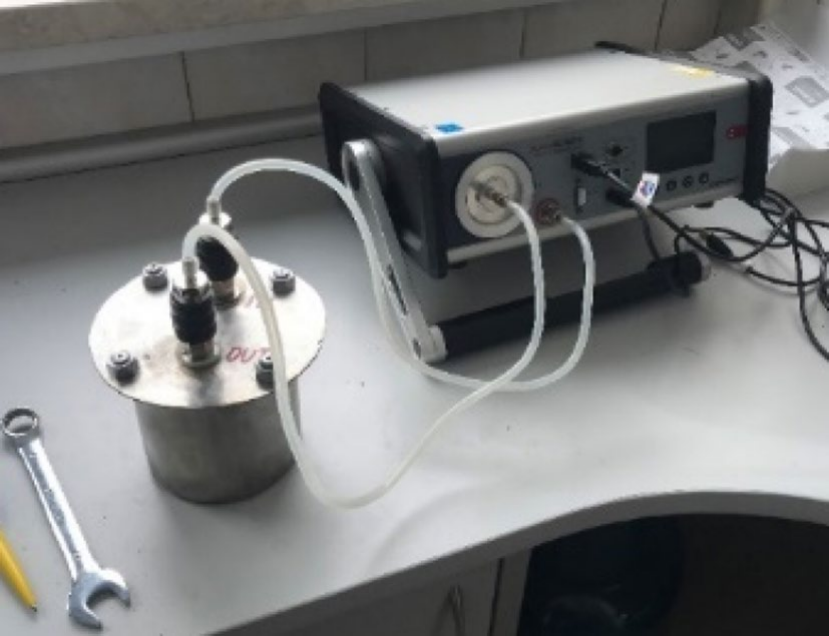
Set up for radon accumulation measurements for assessment of the emanation coefficient in the laboratory environment. Set include the small accumulation chamber with the total volume of 0.00114 m^3^ (present on the left side) operating with the AlphaGuard instrument (presented on the right).

The radon emission from samples in the laboratory environment were measured using an AlphaGUARD instrument equipped with a measuring chamber made of stainless steel, as presented on the left in Fig. 2. The chamber was 11 cm in diameter and 12 cm in height, giving 0.00114 m^3^ of volume. The measurement of the radon concentration in air was made setting a 1 dm^3^ min^−1^ flow rate and 10 min reading cycles. Data were next averaged for 1 h, resulting to one data point represents the average values from six originally measured data points. Measurement for one sample took 20 h. Values of radon concentration in air was registered with the uncertainty for each data point assessed directly by the instrument.

The emanation coefficient ε was determined based on the Ra-226 activity concentration and radon potential Ω according to the methodology proposed by^33^ and making an additional assumption that the samples were dried to zero humidity, which implied that no pores filled with water were present within the samples during measurements. The assumption reduces the equation for calculating the time bound exhalation constant.

The emanation coefficient expressed in % was evaluated according to the equation:

2 $$\varepsilon =\frac{{\Omega }}{{Ac}_{Ra}}\cdot 100{\%}$$

where Ac_Ra_ represents the Ra-226 activity concentration in a soil sample expressed in Bq kg^−1^.

The Ac_Ra_ was assessed using gamma spectrometry and a high purity germanium detector according to the methodology described by^25^. For properly Ra-226 activity concentration in soil assessment the impact of 185.7 keV gammas from U-235 was subtracted after its evaluation basing on the 63.3 keV peak of Th-234.

The main advantage of the proposed method is the ability to assess the emanation coefficient based on short-time measurements (below 24 h) with a cumulative chamber method. Originally, in the methodology developed by^33^, the assessment requires evaluation of Ω according to the formula:

3 $$\Omega = \frac{{a + \lambda _{{eff}} \cdot C_{{Rn}}^{0} }}{{\lambda _{{Rn}} }}~\frac{{V_{e} }}{m}$$

where a (Bq m^−3^ s^−1^) represents the slope of the linear fit of radon exhalation rate data series measured in the cumulative chamber, λ_eff_ is an effective time constant (s^−1^), C_Rn_^0^ is the initial Rn-222 concentration in the accumulation chamber (Bq m^−3^), λ_Rn_ denotes the Rn-222 decay constant, V_e_ describes the effective accumulation volume in the experimental setup (m^3^), and m (kg) is the mass of the sample.

The effective time constant describes the effective time of the presence of radon exhaled within the experimental set up and is the sum of the Rn-222 decay constant λ_Rn_ (s^−1^), the bound exhalation constant λ_b_ (s^−1^) characterizing the sample, and the leakage constant characterizing the accumulation chamber equipment λ_l_ (s^−1^):

4 $${\lambda }_{eff}={\lambda }_{Rn}+{\lambda }_{b}+{\lambda }_{l}$$

The λ_b_ coefficient is dependent on the soil sample porosity according to the simplified equation:

5 $${\lambda }_{b}={\lambda }_{Rn}\eta \frac{{V}_{0}}{{V}_{e}}$$

where η represents the total porosity of the sample, as the assumption of zero humidity of samples during measurements was made, and V_0_ represents the volume of the sample.

λ_l_ was evaluated experimentally by measuring the Rn-222 decay in the empty accumulation chamber system with a natural radon concentration at the starting point. The most significant compound of total uncertainty for the emanation coefficient was associated with estimation for slope a, C_Rn_^0^ and with assessment of the total porosity for the samples η. The uncertainty was evaluated using differentiation method.

### Radon exhalation rate assessment

**Figure 3:**
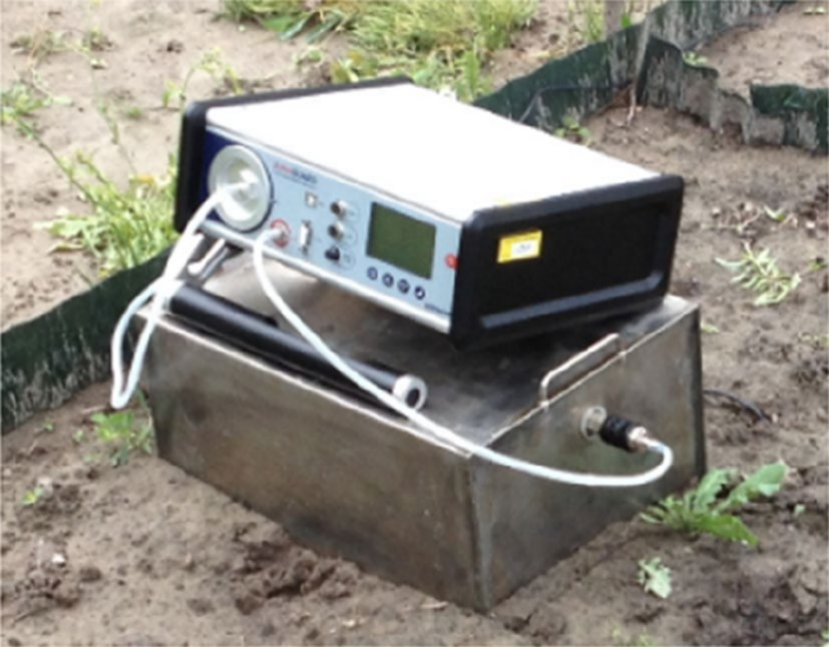
Setup for assessment of the radon exhalation rate in the field measurements. The set consist of the accumulation chamber with 0.024 m^3^ in volume and the AlphaGuard instrument.

The radon exhalation rate (E_Rn_) was assessed according to the methodology presented in^25^. The field radon exhalation rate was assessed indirectly by measurement of radon concentration in air using an AlphaGUARD instrument equipped with accumulation open-wall chamber placed on the ground as presented on the Fig. 3. The chamber volume was 0.024 m^3^. The setup measuring parameters were the same as in the case of the laboratory measurements with the small closed chamber but the measuring time was 70 min giving seven data points for each field. Values of radon concentration in air was registered with the uncertainty for each data point assessed directly by the instrument.

The increase of radon concentration in air (C_Rn_) in accumulation box were registered and the linear function was interpolated basing on seven measuring points registered for each experimental field. Basing on the measurement of radon concentration in air the radon exhalation rate could be assessed according to the equation:

6 $${E}_{Rn}=\frac{V}{A}\frac{\partial {C}_{Rn}}{\partial t}$$

where V/A are the ratio of accumulation chamber volume to area covered by the chamber and is a constant value of 0.2, $\frac{\partial {C}_{Rn}}{\partial t}$ is a change of radon concentration in air registered in accumulation chamber in time t and was represented as a linear fit into the experimental data as:

7 $${C}_{Rn}=a\cdot t+b$$

After differentiation we can compute the radon exhalation rate as slope, a of linear fit scaled by 0.2:

8 $${E}_{Rn}=0.2a$$

The uncertainty for radon exhalation rate assessment was associated mainly with the assessment of the slope of linear fit ad was calculated as a standard deviation for data point used for linear fits.

## Results

**Figure 4:**
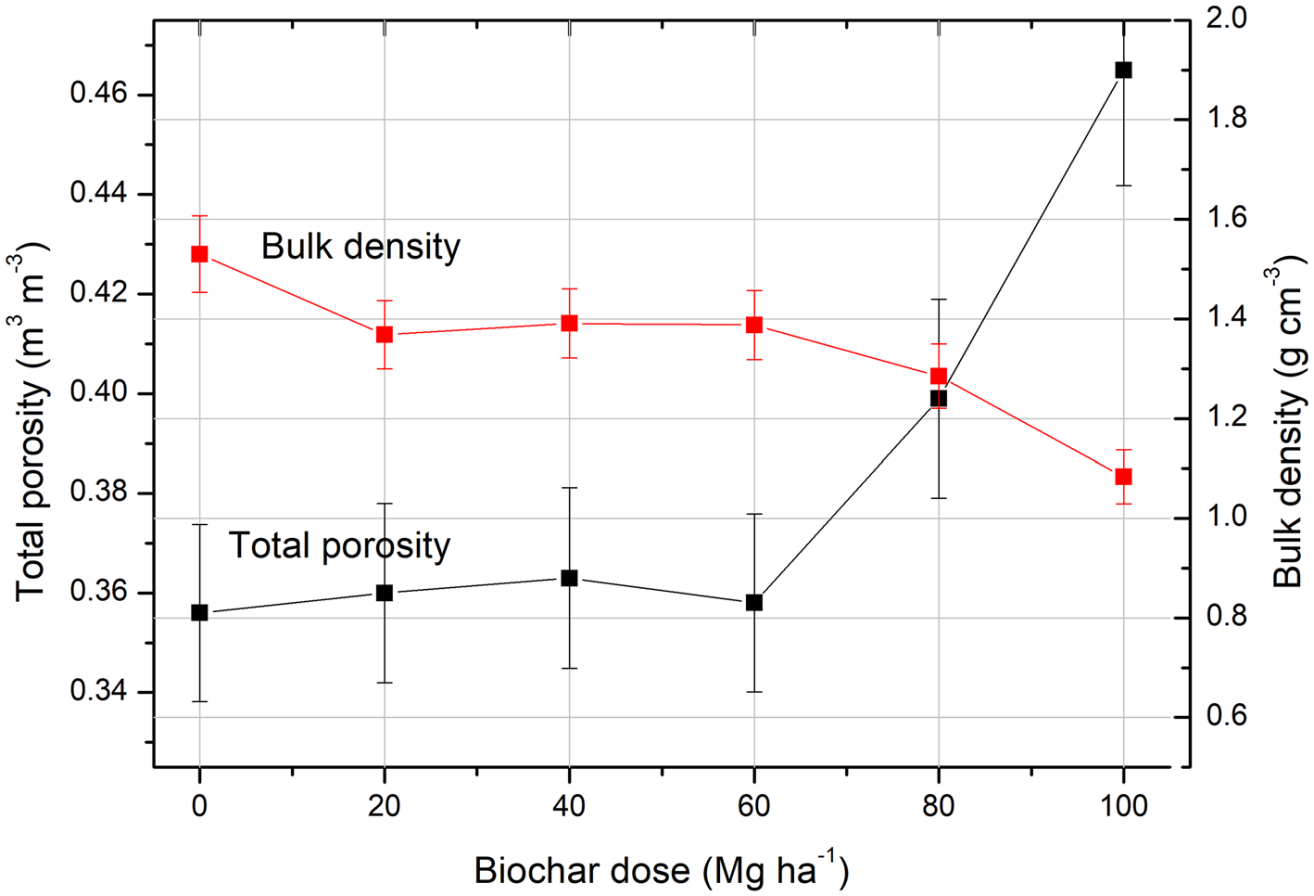
Bulk density (red graph) and total porosity (black graph) with the uncertainties (red and black vertical lines) as a function of the biochar dose applied into the soil. Data for six samples investigated in the laboratory for assessment of the emanation coefficient.

The values of total porosity and bulk density for the samples investigated in the laboratory are presented in Fig. 4. Depending on the biochar dose, the bulk density decreased from 1.51 g cm^−3^ for the sample without biochar to 1.09 g cm^−3^ for the sample supplemented with the 100 Mg ha^−1^ biochar dose, as was presented on Table 1, together with the information on the mass for specific samples.

**Table 1:** Data of bulk density assessment with the information on the soil samples mass, depending on the biochar dose applied into the soil in the experimental fields. Bulk density values presented on the Fig. 4.

Sample no	Biochar dose (Mg ha^−1^)	Soil mass (g)	Bulk density (g cm^−3^)
1	0	153	1.51
2	20	137	1.37
3	40	139	1.39
4	60	138	1.39
5	80	128	1.29
6	100	108	1.09

The total porosity varied from 0.358 m^3^ m^−3^ for the 0 Mg ha^−1^ sample to 0.466 m^3^ m^−3^ for the sample with the 100 Mg ha^−1^ biochar dose.

**Figure 5:**
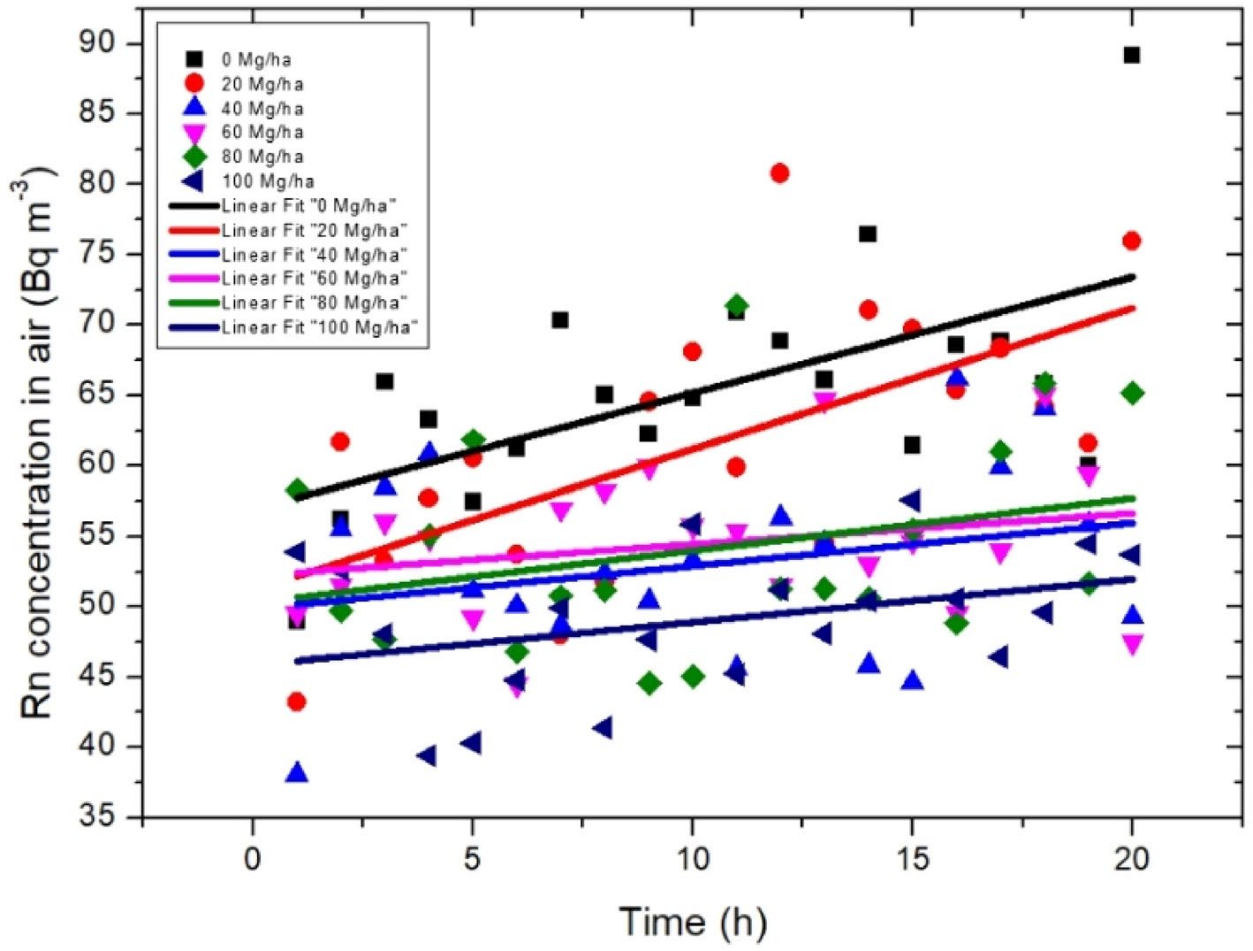
Radon concentration in air measured for five soil samples collected in the experimental fields and for the control sample (0 Mg ha^−1^) as a function of the measurement time. Symbols represent measurements results from the AlphaGUARD instrument, and the lines represent the linear fits. The biochar dose applied into the soil ranged from 20 to 100 Mg ha^−1^.

The results of radon accumulation in the small closed chamber during the laboratory measurements for samples collected from the experimental fields and investigated in terms of bulk density and total porosity are presented in Fig. 5. The highest value of the radon concentration of 90 Bq m^−3^ was observed for the control sample. In general, the lowest values were noted for the sample collected from the field treated with the 100 Mg ha^−1^ biochar dose. The results for samples collected in the fields with the biochar dose of 40, 60, and 80 Mg ha^−1^ were at the same level as those observed at linear fitting.

**Figure 6:**
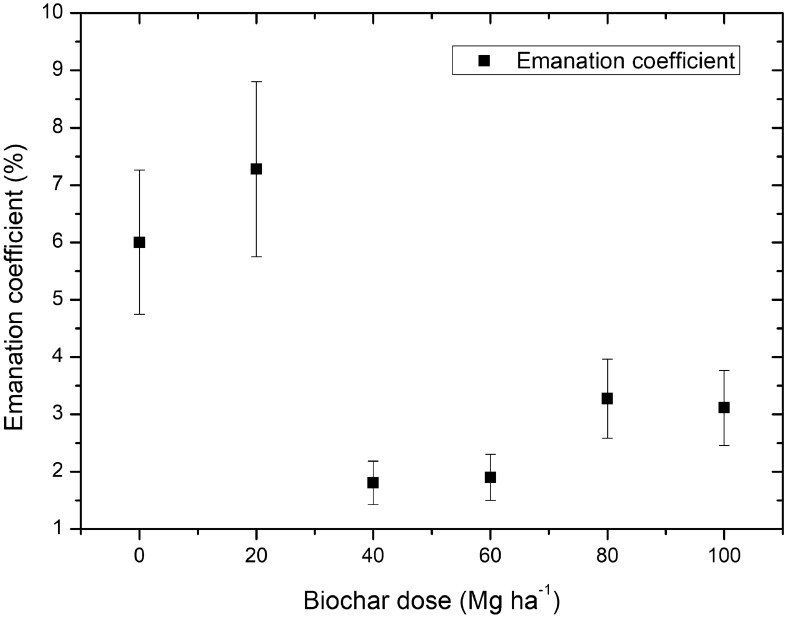
Emanation coefficient with the uncertainty values, expressed as % of radon particles forced into the free air-filled space in soil samples as a function of the biochar dose applied into the soil.

The values of the emanation coefficient calculated using Eq. (2) are presented in Fig. 6. The highest emanation coefficient of 7.28% was observed for the sample treated with the 20 Mg ha^−1^ biochar dose, whereas and the lowest value of 1.81% was calculated for the sample collected from the field supplemented with 40 Mg ha^−1^. The values of all parameters, including the slopes for linear fits presented in Fig. 5 and used in the calculation process for emanation coefficient, were presented in Table 2.

**Table 2:** Parameters values used for calculation a emanation coefficient incorporating Eqs. (2–5) and the obtained values of ε with the total uncertainty u(ε).

Biochar dose (Mg ha^−1^)	Sample mass, m (kg)	Total porosity, η (m^3^ m^−3^)	Effective decay constant λ_eff_ (s^−1^)	Slope, a	Ra-226 activity concentration, Ac_Ra_ (Bq kg^−1^)	Emanation coefficient, ε ± u(ε) (%)
0	0.149	0.356	0.02426	0.138	20.7	6.00 ± 0.99
20	0.132	0.360	0.02426	0.167	23.5	7.28 ± 1.24
40	0.139	0.363	0.02426	0.051	27.8	1.81 ± 0.28
60	0.136	0.358	0.02426	0.037	19.7	1.90 ± 0.37
80	0.116	0.399	0.02429	0.062	22.1	3.27 ± 0.72
100	0.110	0.465	0.02434	0.051	20.3	3.11 ± 0.73

**Figure 7:**
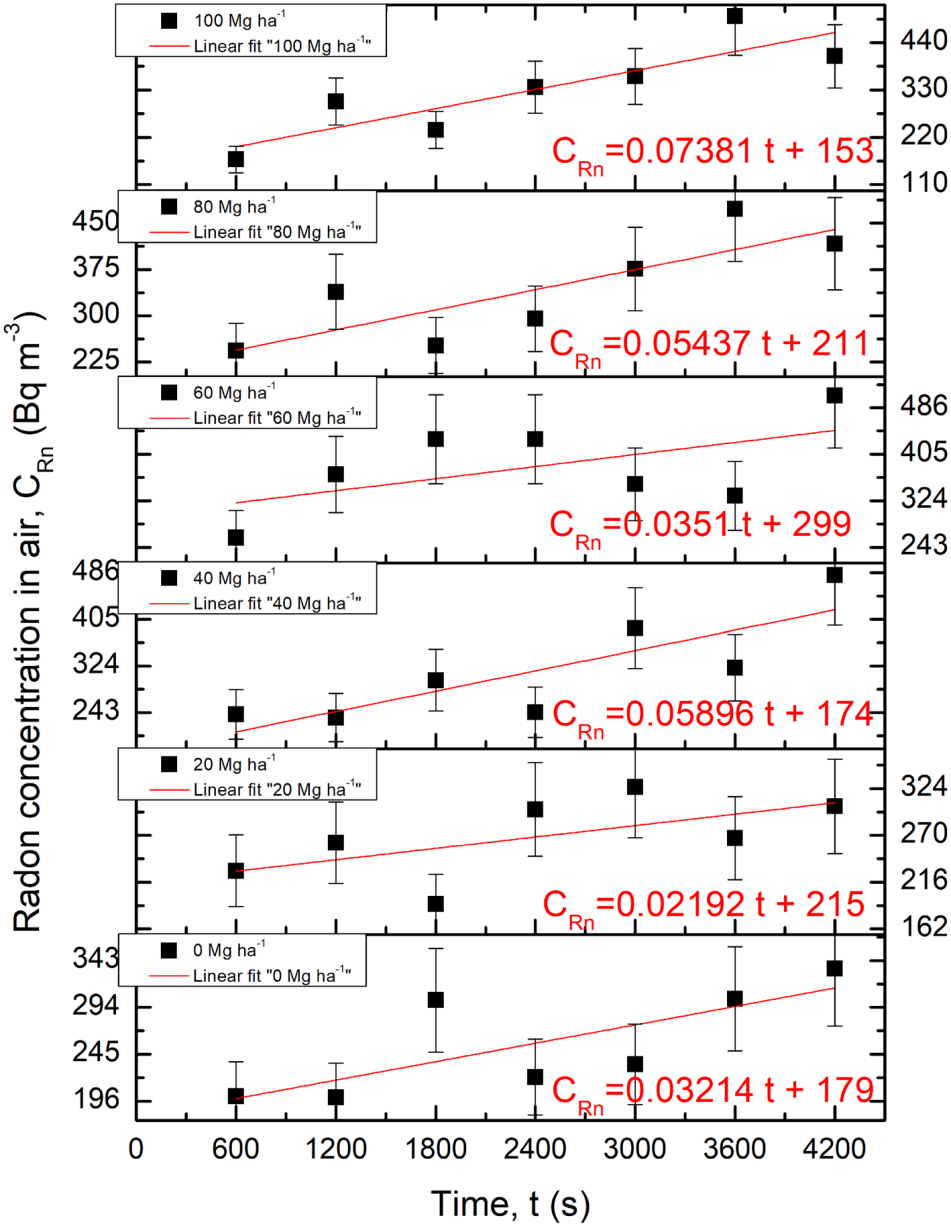
Results of radon accumulation expressed as the radon concentration in air (black squares) as a function of time for five investigated experimental fields and for the control field (denoted as 0 Mg ha^−1^). The linear fitting is presented as red lines and in the form of linear equations (in red). Errors presented for data points were calculated directly by the AlphaGUARD instrument.

Figure 7 presents raw measurement results from the field experiment. An increase in the radon concentration in air within the accumulation chamber measured as a function of time for the particular fields: five with applied biochar and one without biochar is presented. The red line expresses the linear fits represented by the linear equation also shown for each examined field. The lowest slope was observed for the filed where 20 Mg ha^−1^ biochar dose was applied and the highest one for the field were the biochar was applied at the dose of 100 Mg ha^−1^.

**Figure 8:**
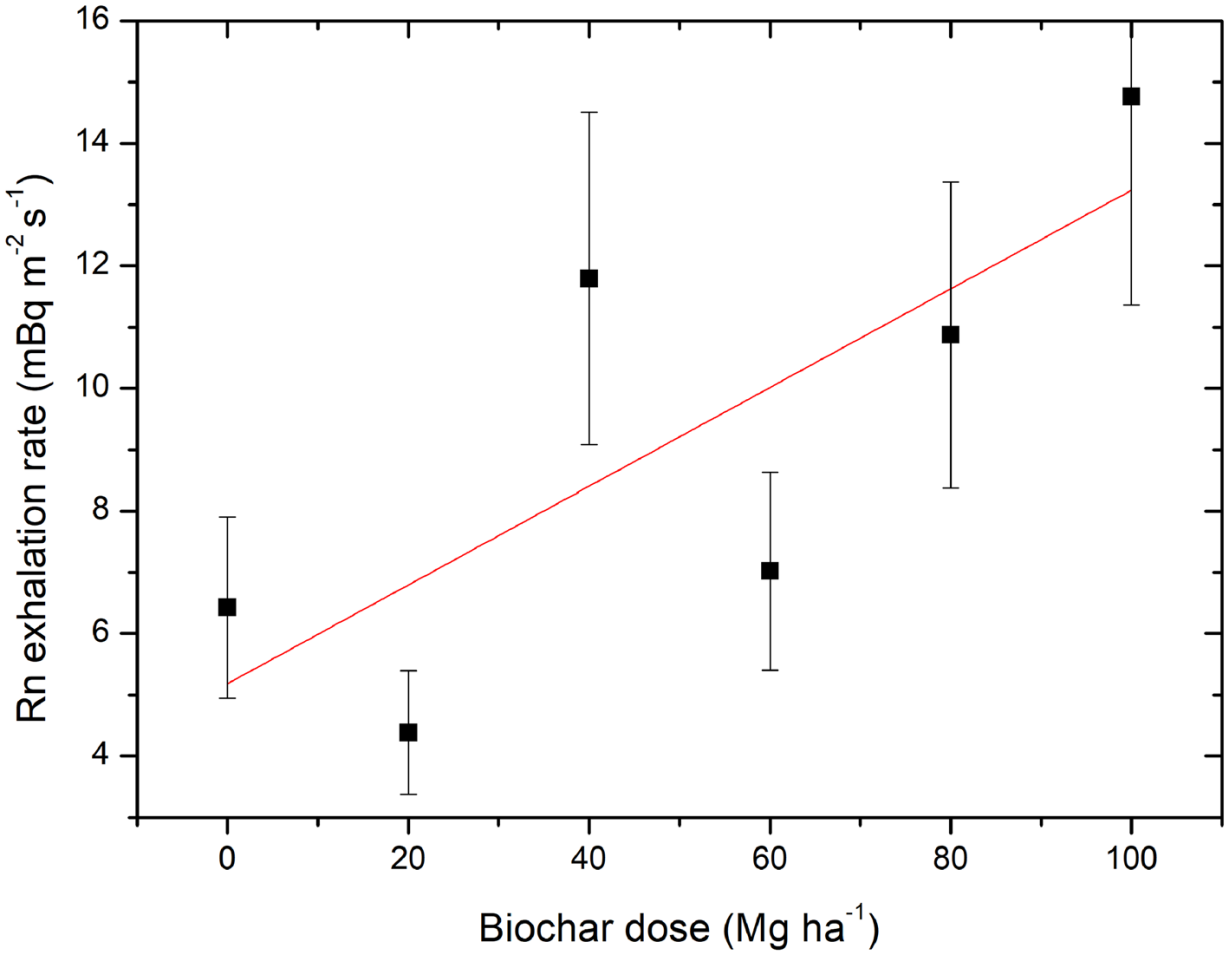
Radon exhalation rate (black squares) with uncertainties observed for five experimental fields and for the control one as a function of the biochar dose applied into the soil. The red line represents the increased trend associated with the results.

Based on the slopes of the linear equations presented in Fig. 7, the radon exhalation rates were calculated according to the Eq. (8). The results for radon exhalation rate including the uncertainties are presented in Fig. 8. The lowest exhalation was 4.4 mBq m^−2^ s^−1^ and the highest value observed for the 100 Mg ha^−1^ biochar dose was 14.8 mBq m^−2^ s^−1^. The maximum uncertainty for assessed radon exhalation rate was obtained for field with 60 Mg ha^−1^ and reached 65%. The lowest uncertainty of radon exhalation rate was registered for field with 100 Mg ha^−1^ biochar dose and was 25%.

## Discussion

Many aspect of biochar application into the soil were already conducted in research. The actual published data concerning impact of biochar application into the soil were mainly focused on the investigation of physic-chemical properties of amendment soil.

As demonstrated before^25^ and confirmed by the present results, the application of biochar into the soil had a considerable effect on the radon exhalation process and in more general for radiological state of the natural environment. The present results show that the increase in the biochar dose applied into the soil from 0 to 100 Mg ha^−1^ contributed to the increase in the radon exhalation rate by approx. 10 mBq m^−2^ s^−1^. The result is in good agreement with a previously reported value^25^, where the increase was at the level of 9 mBq m^−2^ s^−1^.

**Figure 9:**
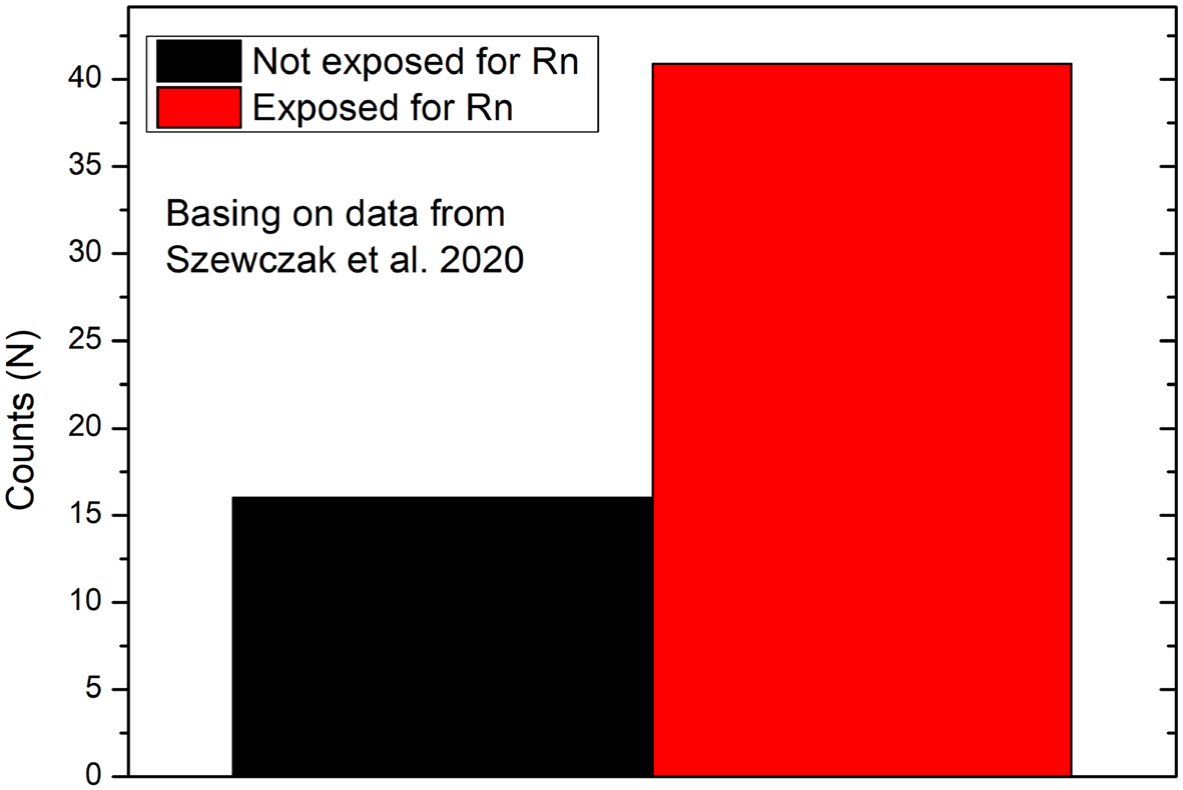
Averaged number of counts registered in liquid scintillation detector for biochar exposed and not exposed for radon in laboratory condition (basing on the data presented in^25^).

Radon exhalation rate is a complex process, where the soil structure and soil composition^10,34^ as well as a soil moisture^35^ plays important role. In context of the soil structure the higher exhalation rate is associated with higher number of free pores present within the soil volume. From the other site the exhalation rate will decrease with higher soil moisture as pores filled by water are impassable for moving radon particles. Considering the impact of biochar for soil properties we can observe that biochar causes increase in the soil porosity as was presented on Fig. 5, and as a consequences making more free paths that radon could escape into the soil surface. On the other site it was proved that biochar influencing on water retention of soil. Therefore more pores filled with water are present in soil after biochar application, a specially in the environmental condition. Moreover we should identify another phenomena governing the radon movement within the soil after biochar application. The biochar by its high total surface area was identified as a good absorber for gases including radon, see Fig. 9. As a result after biochar application into the soil the increased soil self-absorption for radon is expected. These three phenomena have been identified as the main causes of the radon exhalation rate changes after biochar application into the soil.

Basing on the results of exhalation rate assessment it should been concluded that the dominant effect on the change in radon exhalation rate was related to the increase in soil porosity as a result of the biochar application. The effect of increased water content in soil and increased self-absorption of soil after biochar application have a less important impact for exhalation. However, the most significant increase in total porosity were observed in the sample treated with the 80 Mg ha^−1^ biochar dose. The bulk density for samples amended with 20–60 Mg ha^−1^ biochar had a stable value. In addition, considering the values of total porosity, quite stable values were observed for samples supplemented with doses from 0 to 60 Mg ha^−1^.

It is known that the radon emanation is mainly related to the structure of the soil and the total specific surface of the sample^10^. The results of the emanation coefficient assessment indicate dependence of the coefficient on the biochar dose applied into the soil. The most visible decrease in emanation was observed between samples treated with 20 and 40 Mg ha^−1^ with a value of 7.28% and 1.81%, respectively. The results show that application of 20 Mg ha^−1^ of biochar does not exert a considerable impact in the emanation process. As the emanation coefficient were assessed in laboratory condition after drying the soil samples the decrease in emanation was associated with the self-absorption of soil by presence of biochar particles.

## Conclusions

Biochar application into the soil has a considerable impact on the radon emanation process as well as on the exhalation rate of radon on the soil surface. Depending on the dose of biochar applied into the soil, the emanation coefficient was reduced from more than 7% to less than 2%, at the same time causing a observable increase in the radon exhalation rate. Given the increasing trend of using biochar in modern agriculture, impact of this material on the radiological state of the soil and environment should be considered. The presented results should be treated as a starting point for profound research on the impact of biochar on environmental radioactivity in the wider range.
